# Restoring heart function and electrical integrity: closing the circuit

**DOI:** 10.1038/s41536-017-0015-2

**Published:** 2017-04-07

**Authors:** Luís Miguel Monteiro, Francisco Vasques-Nóvoa, Lino Ferreira, Perpétua Pinto-do-Ó, Diana Santos Nascimento

**Affiliations:** 1 i3S—Instituto de Investigação e Inovação em Saúde, Universidade do Porto, Porto, Portugal; 2 INEB—Instituto de Engenharia Biomédica, Universidade do Porto, Porto, Portugal; 3 CNC—Center for Neuroscience and Cell Biology, Universidade de Coimbra, Coimbra, Portugal; 4 Departamento de Fisiologia e Cirurgia Cardiotorácica, Faculdade de Medicina da Universidade do Porto, Porto, Portugal; 5 ICBAS—Instituto de Ciências Biomédicas Abel Salazar, Universidade do Porto, Porto, Portugal

## Abstract

Cardiovascular diseases are the main cause of death in the world and are often associated with the occurrence of arrhythmias due to disruption of myocardial electrical integrity. Pathologies involving dysfunction of the specialized cardiac excitatory/conductive tissue are also common and constitute an added source of morbidity and mortality since current standard therapies withstand a great number of limitations. As electrical integrity is essential for a well-functioning heart, innovative strategies have been bioengineered to improve heart conduction and/or promote myocardial repair, based on: (1) gene and/or cell delivery; or (2) conductive biomaterials as tools for cardiac tissue engineering. Herein we aim to review the *state-of-art* in the area, while briefly describing the biological principles underlying the heart electrical/conduction system and how this system can be disrupted in heart disease. Suggestions regarding targets for future studies are also presented.

## Introduction

Cardiovascular diseases (CVD) are the leading cause of mortality worldwide with current estimation of 17.3 million deaths per year and with an expected increase up to 23.6 million deaths by the year 2030, representing 31% of all global deaths.^1^ Importantly, CVD risk factors promote cardiac structural changes that are frequently associated to electrical disruption and the onset of arrhythmias,^2^ which account for approximately 50% of deaths associated with chronic heart failure.^3^ In addition, CVD may also encompass the impairment of the cardiac conduction system itself.

Aiming to promote cardiac tissue repair/regeneration and/or reduce disease symptoms, while surpassing the various shortcomings of the current gold-standard approaches (e.g., drugs, electronic pacemakers, implantable cardioverter defibrillators, heart transplantation), innovative therapeutic strategies have been emerging. The latter either focuses on the improvement of heart function in pathological scenarios involving dysfunction/loss of working cardiomyocytes (CMs), as is the case of acute myocardial infarction (MI) (reviewed in ref. 4); or are intended to decrease the occurrence of arrhythmias and/or to restore the disrupted cardiac conduction or action potential (AP) (reviewed in ref. 5). Although these two different approaches have been extensively reviewed separately, integrative reviews are lacking. Acknowledging the importance of cardiac conduction for the proper restoration of CM contractility and myocardial function, we herein provide a concise overview of the state-of-art on novel strategies to restore electrical conduction and to promote myocardial repair by improving electrical coupling of implanted cells and/or biomaterials with the native myocardial tissue. In addition, along this revision a brief description of the biological basis of the cardiac electrical conduction system and its subsequent disruption in pathological situations is presented.

## Cardiac electrical system

A close interaction between specialized excitatory and conductive components and the working CMs (contractile component) is essential for the successive and rhythmic contractions and relaxations of the myocardium, which promote unidirectional blood flow at an adequate pressure.

**Fig. 1:**
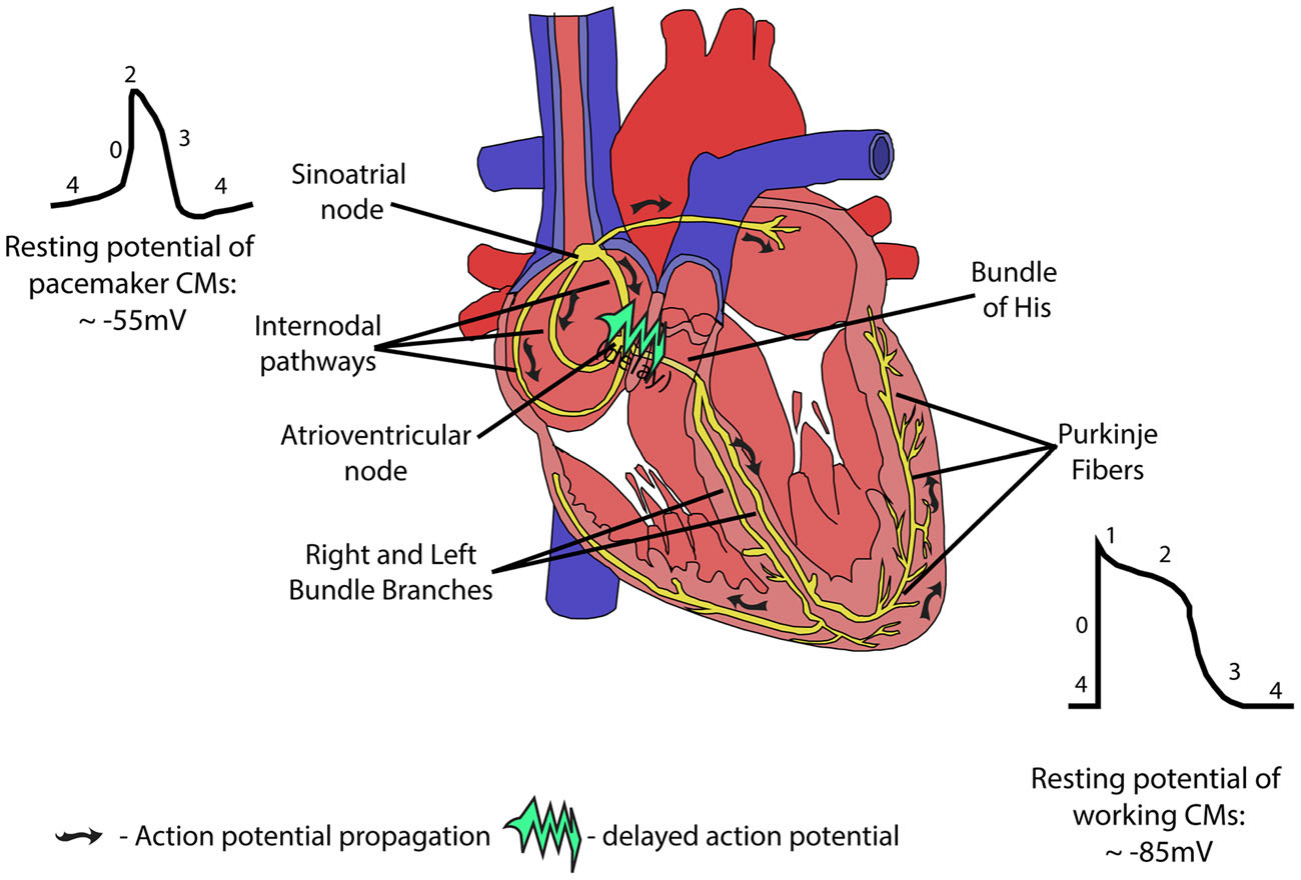
Representation of the anatomy of the cardiac conduction system and the path of the action potential propagation (*arrows*) including the time delay observed at the AV junction (*green arrow*). The shape of the AP on SAN (*upper left*) and working myocytes (*lower right*) are represented along with the respective resting potentials and the different phases of the signal (numbers)

The main elements of the excitatory and conductive components are the sinoatrial node (SAN), the internodal pathways, the atrioventricular node (AVN), the bundle of His and the Purkinje fibers^6^ (Fig. 1). This system is mainly composed of specialized CMs whose cytoarchitecture and electrophysiological properties vary according to their specific function and differ from working a trial and ventricular CMs.^6^

Specialized CMs of the SAN, regulated by sympathetic and parasympathetic stimuli, spontaneously generate AP that directly propagates to: (1) the atrial myocardium leading to its contraction; (2) the internodal pathways; and, ultimately, to (3) the AVN. At the latter, the impulse propagation suffers an essential delay in order that blood from the atria fills the ventricles before ventricular contraction. Finally, the AP is propagated rapidly through the bundle of His and Purkinje fibers towards the ventricular working myocardium, which then contracts in syncytial manner^7^ (Fig. 1). At the cellular level, cardiac AP represents the variations of the CM membrane potential that follow an initial depolarization from a resting potential (−85 mV in working CMs) to a threshold potential (−40 mV), in a sequence of events mediated by ion channels. Essentially, in working CMs this AP can be divided in five phases (Fig. 1): (a) phase 0: upon stimulation from neighboring cells and depolarization to −40 mV, membrane voltage-gated fast Na^+^ channels (Na_V_1.5 channels) open which causes a rapid intake of Na^+^ ions (I_Na_ currents) triggering a further depolarization to a peak of, approximately, + 20 mV (ref. 7–10); (b) phase 1: Na_V_1.5 channels close and K^+^ channels (e.g., K_V_4.2, K_V_4.3) open leading to a transient outward K^+^ current (I_to_) and, consequently, to a transient repolarization^7, 9, 10^; (c) phase 2: opening of slow L-type calcium inward channels (Ca_V_1.2) concomitant with the current mediated by rapidly (I_Kr_ currents) and slowly (I_Ks_ currents) activating delayed outward rectifying K^+^ channels (e.g., K_V_11.1 and K_V_7.1, respectively), maintain the AP in a relatively constant depolarized state (plateau)^7, 9–11^; (d) phase 3: Ca_V_1.2 channels close and I_Kr_ and I_Ks_ cause a rapid repolarization to the resting potential^7, 9, 10^; (e) phase 4: the membrane potential is maintained at a resting level by K^+^ inward rectifier channels (e.g., K_IR_ 2.1) (I_K1_ currents), Na^+^/K^+^ exchange pumps and Na^+^/Ca^2+^ exchange pumps.^7, 9, 10^ Although working CMs need to be depolarized by neighboring cells, specialized CMs of the SANs and AVNs and Purkinje fibers, the so-called pacemaker cells, are able to spontaneously generate AP. This property is closely related to an unstable, and less negative resting potential (around − 55 mV) comparing to working CMs. Such less negative resting potential is due to a lack of I_K1_ currents and to an increased leakage of Na^+^ resulting in an inward current of these ions (the pacemaker current, I_f_) and that is mediated by the hyperpolarization-activated, cyclic-nucleotide-gated (HCN) channels.^12^ This I_f_ current, and the fact that fastest Na^+^ channels are closed at −55 mV or higher, result on a slow diastolic depolarization of pacemaker cells (phase 4). When membrane potential reaches a threshold level, T-type (e.g., Ca_V_3.1) and slow L-type Ca^2+^ channels open, depolarizing the cell up to + 20 mV (phase 0) (Fig. 1).^7, 10^

The cardiac AP triggers contraction of working CM and successive contraction of the atria and ventricles through a process denominated excitation-contraction coupling. Depolarization of sarcolemma induces opening of L-type Ca^2+^channels and, the subsequent increase in intracellular Ca^2+^ concentration, activates the ryanodine receptors (which are intracellular Ca^2+^ channels) at the membrane of the sarcoplasmic reticulum leading to release of this ion from the sarcoplasmic reticulum to the sarcoplasm, further increasing Ca^2+^ intracellular concentration. These ions bind to subunit C of troponin which causes a conformational change freeing the actin’s myosin-binding sites from tropomyosin, leading to sarcomere contraction. Finally, Ca^2+^ baseline intracellular levels are restored mainly being pumped back to the sarcoplasmic reticulum through the sarcoplasmic reticulum Ca^2+^ ATPase 2a (SERCA2a) or released from the cell via Na^+^/Ca^2+^-exchangers. There are evidences that, acting in late phase of the diastolic depolarization in pacemaker cells, this Ca^2+^ cycling also promotes spontaneous beating (the “calcium clock”), acting in combination with the membrane potential instability and I_f_ current.^13^

Impulse propagation between CM is dependent on intercellular electrical coupling i.e., the ability of one cell to transport ions directly from its sarcoplasm to a neighboring one. This coupling is predominantly achieved through gap junctions which consist in two exactly aligned hemichannels, one from each coupling cell, composed of six subunits of connexin (Cx) proteins. Cx45, Cx40, and Cx43 are the predominant isoforms expressed in CMs.^14^ Cx43 is the predominant isoform of adult working CMs,^15, 16^ whereas Cx40 is expressed in His-Purkinje fibers and atrial working CMs (but not in ventricular working CMs),^15–18^ and Cx45 expression is predominantly found at the AVN.^16, 19^ These three Cx isoforms display different levels of conductance: Cx40 has the highest conductance and Cx45 the lowest.^20^ Interestingly, these characteristics correlate with the velocity of impulse propagation characteristic of the different structures i.e., his-Purkinje fibers are fast-conducting structures, while the AVN is associated with a time delay in AP propagation, however, this effect is also potentiated by smaller and less abundant gap junctions.^19, 21^ Despite the widely spread classical view that impulse propagation between CM occurs primarily through gap junctions (electrotonic model), there are evidences that intercalated discs may actively influence cell–cell impulse transmission, involving differential Na_V_1.5 channel expression and extracellular space charge variations (ephaptic model) reviewed in ref. 22).

## Disruption of electrical conduction in heart diseases

Cardiac electrical disruption often results in arrhythmias involving altered heart rates (bradyarrhythmias and tachyarrhythmias corresponding to low and high heart rates, respectively), premature beats, atrial flutter and fibrillations, which comprise an unorganized AP propagation through the myocardial mass resulting in uncoordinated contractions and relaxations between different regions of the myocardium, which can be supraventricular (e.g., atrial fibrillation) or ventricular.^5^

Tachyarrhythmias and fibrillations are frequently associated with CVDs, especially in a heart failure scenarios. The arrhythmogenic properties underlying heart failure are due to different factors, namely: ion channel remodeling; reduced excitability; impaired Ca^2+^ cycling; decreased intercellular electrical coupling; and formation of electrically isolating fibrotic tissue.^2, 23^ Concerning ion channel remodeling, evidences show that, upon heart failure, there is a reduction on the expression of ion channels related to repolarization currents (e.g., I_to_, I_Ks_) with concomitant increase in a delayed inward Na^+^ current. These events result in prolonged AP duration, which promotes the occurrence of Ca^2+^-mediated after-depolarizations. Reduced excitability, for instance during acute MI, usually occurs due to different factors such as increased K^+^ extracellular concentration. The main effect of the latter is depolarization of CM resting potential, which induces a partial inactivation of the voltage-gated Na^+^, reducing I_Na_ current and, consequently, excitability and conduction velocities. Reduced conduction velocities promote the onset of reentrant arrhythmias, as discussed below in this section. Calcium cycling is also commonly affected during heart failure, for instance, increased diastolic intracellular Ca^2+^ concentration, mainly due to higher leakage from the sarcoplasmic reticulum (a consequence of impaired ryanodine receptor function) and/or decreased reuptake of this ions from the sarcoplasmic reticulum (due to SERCA2a defective activity), are observed. This increase on the diastolic concentration also triggers after-depolarizations.^23, 24^ Concerning intercellular electrical coupling, Cx43 expression is reduced up to 50% in heart failure.^25^ Apart from causing conduction deceleration and discontinuity, defective intercellular coupling between the CM also results in increased subthreshold depolarization, which slowly inactivates the voltage-gated Na^+^ channels, further reducing the I_Na_ current and excitability.^2, 23^ Furthermore, deposition of collagenous scar tissue, which is an electrical insulator, is common to a wide number of cardiovascular disorders and is particular evident after MI. Collagen deposition results on electrically isolated fibers of viable myocardium, discontinuing the conduction path and globally reducing the AP propagation velocity and consequently promoting the onset of reentrant arrhythmias.^2, 23^

The most common phenomenon underlying the maintenance of ventricular and supraventricular (e.g., atrial fibrillation) tachyarrhythmia and fibrillations is the “reentry” mechanism. This can occur in a region of the myocardium in which AP encounters a path divided in two, a fast pathway (normal) and a slow pathway (for instance, a myocardial network containing scar tissue), and which converge again in a downstream position of the path. This situation can lead to the creation of successive AP propagation cycles, in which AP propagates in circles, around the referred fast and slow pathways, and also backwards, increasing the frequency of excitation and consequently the heart rate. One pre-requisite for an anatomical reentry cycle to be sustained is the wavelength of the signal (defined as the product between the conduction velocity and the duration of refractory state) to be shorter than the physical, anatomical path in which the cycle occurs.^2, 5, 23^

The most relevant therapies that are available nowadays for tachyarrhythmias are: (i) anti-arrhythmic drugs, which still have low efficacy and can, in specific circumstances, further aggravate the disorder^26^; (ii) implantable cardioverter defibrillators, which only act upon the onset of arrhythmias and display the limitations of implantable devices; and (iii) invasive procedures such as atrial flutter/fibrillation ablation, which consists in permanently disrupting the atrial reentrant pathway or isolating atrial fibrillation trigger points, therefore, protecting the ventricles from the arrhythmic signal originated in the atria.

## Novel strategies to restore myocardial electrical conduction

**Fig. 2:**
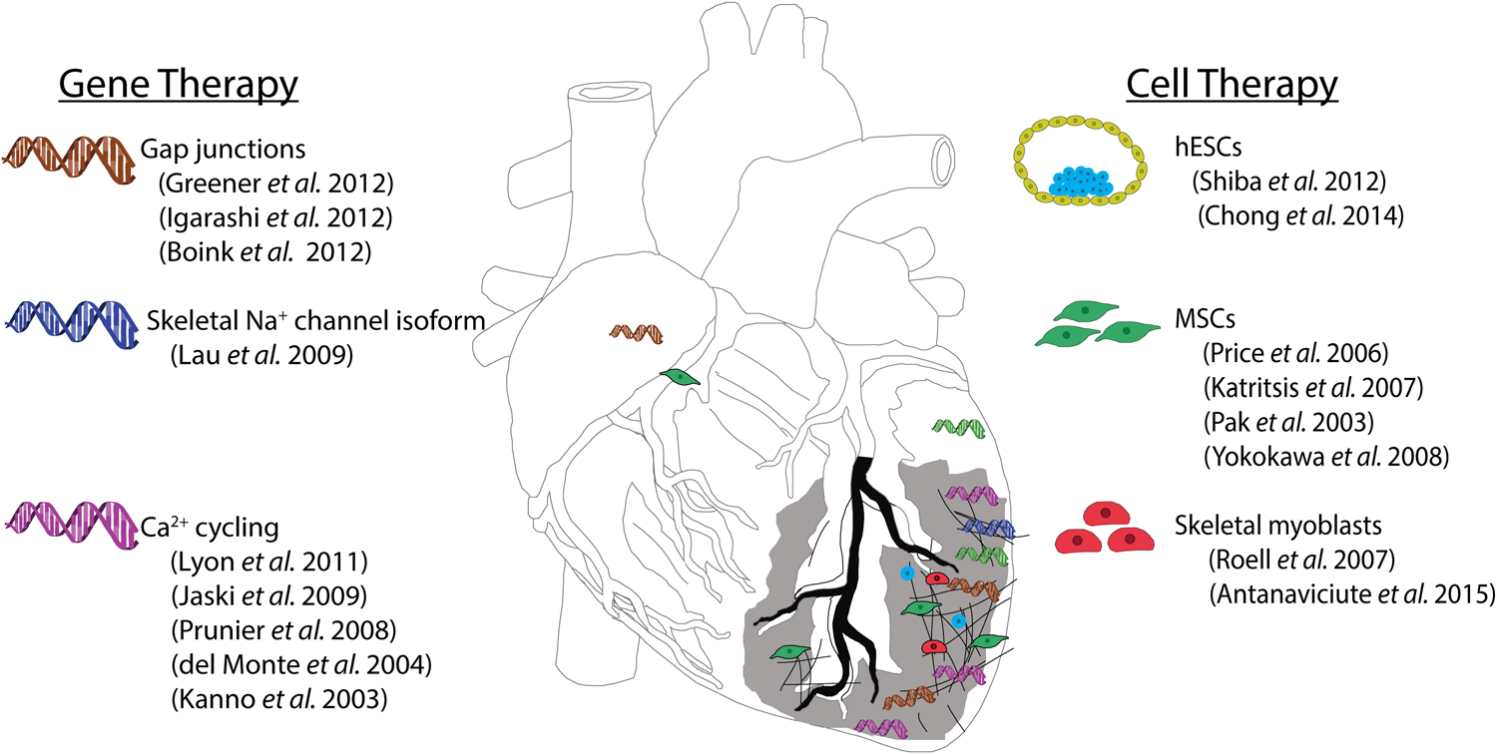
Scheme representing the discussed gene and cell delivery strategies for altering cardiac conduction, along with the delivery sites. A diseased heart with reduced electrical integrity is represented. Icons positioned on the *gray* region are indicative of studies involving MI animal models. The cell sources and gene therapies strategies are represented on the side columns

Functionally proficient cardiac electrical coupling highly depends on molecular players (e.g., ion channels, gap junctions) thus, gene-related therapies along with targeted delivery, hold high potential for restoring cardiac electrophysiology. In addition, the potential of cell therapies to modulate cardiac electrical integrity has also been under recent scrutiny (Fig. 2).

Aiming to assess the effects of said approaches on myocardial electrical conduction, different electrophysiological evaluation methods can be applied. Surface electrocardiography (ECG) and electrophysiological studies (EPSs) (which generally involve the use of intracardiac electrodes) are an example of methods borrowed from the clinical practice. Surface ECGs, the gold-standard for evaluating cardiac electrical activity, are simple, non-invasive procedures in which the electrical activity of the heart is detected by electrodes placed over the skin. ECGs allow the detection of cardiac arrhythmias and are compatible with continuous monitoring, thus allowing the detection of sporadic arrhythmic events that would be difficult to detect in a short period of time. However, although surface ECGs can be measured at different positions (ECG leads), these only allow the measurement of the electrical activity of the heart as a whole, thus not providing a precise, local information of the myocardial electrical conduction and are less amenable to assess local arrhythmogenicity or the mechanisms underlying observed arrhythmias. Aiming to surpass these limitations, the more invasive EPSs can be applied. In brief, electrode catheters can be inserted percutaneously through a vein (e.g., femoral vein) and then placed inside the heart cavities to locally measure electrical signals, resorting or not to local electrical stimulation to evaluate, for instance, arrhythmia inducibility. However, since the mechanisms associated with arrhythmias are often related with myocardial tissue structural disruption and/or spatial changes in AP parameters (e.g., duration, refractoriness), techniques with a higher spatiotemporal resolution should be used, as it is the case of the cardiac optical mapping.^27, 28^ At the moment, this method has only been applied in the context of animal research as is applied in Langerdoff-perfused hearts, where the coronary arteries are perfused, in a retrograde manner, with oxygenated Tyrode’s solution mixed with voltage-sensitive and/or calcium-sensitive fluorescent dyes. Fluorescence signals are then acquired by high-speed charge-coupled-device cameras to visualize and analyze AP propagation while knowing exactly the sites where the electrophysiological changes are occurring.^27, 28^ Moreover, developments have been made regarding the possibility to apply cardiac optical mapping with in an in situ, clinically-compatible, manner.^28^ These techniques are essential to assess whether new therapeutic approaches improve local cardiac conduction but also if, in the case of cell therapy, cells are electrically coupled with the native myocardium. For that purpose, studies generally assess the spatial pattern of Cx43 expression by immunofluorescence, which does not necessarily correlate with functional proficiency. Nevertheless, Laflamme and co-workers used a system where embryonic stem cells (ESC)-derived CM (ESC-CM) genetically encoding a fluorescent calcium sensor were injected in guinea pigs^29^ or macaques,^30^ which showed that calcium transients of the transplanted cell construct were synchronized with surface ECG signals.

### Gene therapies to treat arrhythmias

Gene therapy approaches for treating or reducing the symptoms of ventricular or supraventricular tachyarrhythmias or fibrillations essentially involve: (1) direct repair of intercellular conduction—mainly by overexpressing connexins^2, 31–33^; (2) modulation of AP characteristics—such as increasing the AP upstroke velocity or altering AP duration or refractory period^34–36^; and (3) restoration of calcium cycling—predominantly by upregulating SERCA2a.^37–39^

In most studies, the effects of gene therapies have been demonstrated in animal models of MI and atrial fibrillation. In general, these strategies resulted in a reduced rate of occurrence of ventricular arrhythmias; reduced ventricular arrhythmia inducibility, assessed by programmed electrical stimulation of the myocardium and ECGs; and increased conduction velocities, observed through ex vivo optical mapping and in vivo invasive electrograms. Of note, restoration of Ca^2+^ cycling through SERCA2a overexpression has been applied not only in animal models, but also in humans, particularly in the calcium-up-regulation by percutaneous administration of gene therapy in cardiac disease (CUPID) phase 1/2 clinical trial.^40, 41^ In this trial, recombinant adeno-associated viral vectors (AAV) encoding for the human SERCA2a gene (AAV1/SERCA2a) were injected in patients with advanced heart failure which, during a 12-month follow-up, exhibited reduced symptoms and improved functional status, biomarker profile and left ventricular (LV) function. Additionally, patients presented less cardiovascular events and/or deaths following 3 years, when compared to placebo groups.^41^ However, a recent report showed that when this approach was repeated with a larger number of patients, in a randomized, multinational, double-blinded, placebo-controlled, phase 2b trial (CUPID 2), the AAV1/SERCA2a intracoronary injection did not improve the clinical outcome of patients.^42^ Since no relevant differences were detected between the trial design and patient selection criteria, the observed outcomes were more likely due to viral delivery. For instance, in the CUPID 1 trial a greater proportion of empty viral capsids among total viral particle dose was observed, when compared to the CUPID 2. This could be of relevance, since it was previously shown that empty viral capsids can block the neutralizing activity of the serum antibodies, leaving the transgene-containing capsids free for cellular uptake.^43^ In addition, the authors quantified the AAV1/SERCA2a present in the myocardial tissue of seven patients whose state worsened and found out that the quantity was in the lower threshold for dose–response curves (<500 copies per μg of DNA). Although the number of CMs containing the transgene is unclear (due to the variable ploidy of these cells), this is an evidence that the delivery system might not be efficient enough.

Concerning experimental models, SERCA2a in ischemia-reperfusion (temporary) MI rat and porcine models decreased the number of life-threatening arrhythmias, that effect was not observed or even reversed in permanently occluded rat and porcine models of MI, possibly because Ca^2+^ cell overload and instability are more significant during reperfusion rather than in a lasting ischemic state.^44, 45^ This result shows the importance of testing different ischemic models while aiming at the treatment of a disease such as acute MI. Hence, the etiology of arrhythmias should be carefully considered when developing novel therapeutic solutions. For instance, since AP propagation is dependent on the fast inward Na^+^ currents, one would hypothesize that in heart failure scenarios, in which detrimental ionic channel remodeling occurs, Na_V_1.5 channel overexpression would have potentially restored conduction and preventing the occurrence of reentrant arrhythmias. However, MI causes a general membrane depolarization, leading to Na_V_1.5 channel inactivation which could hinder the usefulness of its overexpression. In that regard, a skeletal Na^+^ channel isoform (Na_V_1.4), which inactivates at a less negative voltage, has been tested. This isoform results in a less extensive AP upstroke in vitro in CMs subjected to a depolarizing medium and has anti-arrhythmic effects in vivo.^34–36^ Connexin-related strategies are an interesting example how one should not overlook the potential detrimental consequences of anti-arrhythmic strategies on the overall homeostasis of the myocardium, in the context of certain disease scenarios. Studies involving connexins overexpression had promising results,^31, 32^ however, it has also been shown that overexpressing Cx32 in a canine MI model causes an increase on the infarct size^33^ by supporting the spread of inflammatory mediators throughout the myocardium.^46^ Thus, although connexins could restore electrical integrity aspects of the myocardium, they could also negatively interfere with the myocardial repair and even have proarrhythmic effects due to increased scarring, reinforcing the notion that strategies should be thought in an integrative manner.

#### Limitations and future directions

Globally, gene therapy constitutes a promising approach, allowing overexpression or silencing of a plethora of molecular players; however, several shortcomings of this strategy can be anticipated.

Firstly, caution is required when selecting the molecular target since, for instance, although in vitro data may support the overexpression of a certain molecular player, the tissue environment subsequent to a particular pathology may result on an ineffective or unexpectedly deleterious overexpression, as in the case of Na_V_1.5 channel and connexins, respectively.

The gene delivery vector must also be wisely selected, and the rationale for this decision is intricately related to the duration of expression, specificity, transduction efficiency, and safety. Most animal studies use adenoviral vectors, which only allow a transient expression (approximately 2 weeks) of the transgene, which is rather limiting. A promising alternative for translational studies are AAV (used in the CUPID trial), which allow an expression that persists for months.^47^ In addition, several serotypes of these vectors (AAV1, AAV6, AAV8, and AAV9) show a significant tropism for transducing CMs in mice,^48^ although the transduction of other myocardial cell types (e.g., endothelial cells and fibroblasts) cannot be ignored. A disadvantage of these vectors is the possibility of triggering the immune system towards the production of neutralizing antibodies, one of the reasons that was raised to explain the negative results CUPID 2 trial, due to a lower proportion of empty capsids in the latter.^42, 43^ Lentiviral vectors allow genomic integration of the transgene and long term expression, however, show low cardiac transduction and have safety issues related to random genome integration which may result, for instance, in insertional oncogenesis. The state-of-art clustered regularly interspaced short palindromic repeats (CRISPR)/Cas9 system is a promising innovative method that allows one to edit the genome at a very specific location, removing particular genes and/or replacing them with a transgene.^49^ This allows the transgene to be integrated into the genome without the risk of causing insertional mutagenesis, since it is not performed randomly.

The delivery method is also a great determinant to transduction efficiency. In the heart, several methods can be adopted for gene constructs delivery, namely: catheter-based intracoronary injections (anterograde or retrograde), direct intramyocardial injections and pericardial delivery. In the CUPID trial, an anterograde intracoronary injection was performed, which, although not among the most efficient methods, is a simple and minimally invasive method suitable for a clinical setting.^42^ Conversely, intramyocardial injection is a local and efficient transgene delivery, however, is highly invasive and the needle may damage the surrounding tissue and have deleterious effects such as arrhythmias.^50^

Another issue to be improved is targeting gene therapy to a specific cell type. In the particular case of the CMs, constructs containing CM-specific promoters such as alpha myosin heavy chain (α-MHC) have been tested, however, these promoters usually result in a relatively low expression when compared with the more ubiquitous, constitutive counterparts (e.g., cytomegalovirus, CMV promoter).^51^

### Role of cell therapies on cardiac electrical integrity

Considering the limitations of gold-standard therapies for CVD and the often deleterious LV tissue remodeling characteristic of myocardial repair, strategies tailored to improve the latter process have been emerging. Cell therapies are one of the most extensively explored approaches, which involve different cell types (e.g., mesenchymal stem/stromal cells (MSC), skeletal myoblasts, ESC-derived CM, cardiac progenitor cells) reviewed in ref. 52. In these studies, especially in animal models, the main assessed outcomes are LV tissue remodeling, neovascularization and cardiac function. In addition, few studies also evaluate whether these therapies have an effect on cardiac electrical integrity. In that sense, part of the studies in which this effect is assessed or that raise pertinent questions, are herein discussed.

Studies involving MSC or bone marrow-derived cells have shown promising results, in both animal models and clinical trials, neovascularization, reduced infarct size and improved LV function.^53–55^ Nevertheless, these cells raised controversy concerning their influence on cardiac electrical integrity and arrhythmogenicity.^56–65^ The authors showing that MSC or bone marrow-derived cells have a pro-arrhythmic potential in vivo^56, 57, 66^ hypothesize that such effect could be a consequence of the electrical unexcitability of these cells, paracrine factors or the accumulation of inflammatory mediators. Although MSC are electrically unexcitable cells, in vitro studies showed that they are capable of repairing conduction block, in neonatal CM cultures, through connexin-mediated coupling.^67, 68^ Conversely, heterocellular electrical coupling may cause reduced AP propagation velocities and gradients of duration of repolarization, which could promote the occurrence of reentry circuits and consequently arrhythmias.^63, 64^ This mechanism is similar to the effect of cardiac fibroblasts on cardiac conduction in a context of fibrosis as these cells undergo direct electrical coupling with CM via gap junctions,^69^ bridging distant unconnected CM.^70^ It is not yet understood whether this “intercellular bridges” are arrhythmogenic substrates or if the electrical bridging has also some beneficial effects by reducing conduction block through scar tissue. Furthermore, MSC-released paracrine factors can also promote disruption of the myocardial electrical integrity not only by altering AP characteristics, ion channel expression and increase re-entry inducibility of CM (assessed by in vitro transwell experiments)^64^; but also by promoting cardiac nerve sprouting and sympathetic hyperinnervation.^57, 66^ Oppositely, other studies indicate that MSC can reduce the electrical disruption in a MI scenario or even exert an anti-arrhythmic effect.^61, 62, 65^ These authors observed that MSC decreased ventricular arrhythmia inducibility, reduced the disruption of gap junction organization in CM and ameliorated the electrical activity of the infarct border zone. Although mechanistic insights are lacking, the authors speculated that MSC-CM gap junction-mediated coupling support AP propagation into the infarcted region, reducing the length of the anatomical conduction path and reducing the incidence of reentry arrhythmias. Additionally, it was suggested that the lack of electrical excitability in combination with the intercellular coupling could not have a significant pro-arrhythmic effect because the number of surviving MSC in the myocardium decreases in few days, being the proportion MSC:CM much lower than in in vitro experiments where arrhythmogenicity was shown. MSC were shown to upregulate Cx43 in the cardiomyocytic HL-1 cell line in vitro^60^ and improve AV conduction on AV-blocked rats^71^ through paracrine mechanisms. Thus, although MSC are electrically unexcitable and incapable of electromechanical coupling with the host myocardium, evidences point to beneficial effects in cardiac function in the absence of side effects. In fact, clinical studies show no conspicuous anti-arrhythmic effect by these cells.^58, 59^

Skeletal myoblasts were among the first cell types applied in animal and clinical studies as cell therapies targeting CVD. This interest mainly stemmed from their capacity to proliferate, increased resistance to ischemia, electrical excitability and the possibility for autologous use.^72^ However, skeletal muscle cells lack expression of connexins upon the formation of myotubes thus exhibiting minimal intercellular coupling. This feature precludes efficient integration of skeletal myoblasts within the myocardium which leads to an increased frequency of arrhythmic events, despite reported positive effects regarding other aspects.^65, 73, 74^ Without intercellular coupling, these cells form clusters electrically isolated from the myocardium, blocking AP propagation in that region, thus rendering the electrical activity of the cells almost irrelevant. To surpass this limitation, some authors overexpressed Cx43 on cultured skeletal myoblasts which improved electrical coupling with CM^75, 76^ further encouraging in vivo tests.^77–81^ Myocardial delivery of these cells in a cryoinjury MI murine model improved electrical coupling between skeletal myoblasts and host CM, with lower incidence of sustained arrhythmias and the ventricular arrhythmia inducibility decreased when compared to regular skeletal myoblast injection.^78^ Conversely, although an amelioration of electrical coupling and improved electrical activity was observed, other studies involving rat^81^ and rabbit^79^ MI models reported that this coupling was insufficient to prevent arrhythmic events and that LV functional improvement was modest. This may relate to distinct AP characteristics of skeletal myoblasts which, in a cardiac environment, may also undergo downregulation of voltage-gated sodium, potassium, and calcium channels,^82^ affecting their excitability and function. Furthermore, as the clinical relevance of skeletal myoblast relies on Cx43 overexpression, alternative non-viral methods of gene expression should be further explored.^80^ One concludes that for cells that are able to survive and proliferate after transplantation into the myocardium, intercellular coupling and electrical and mechanical properties that closely mimic native CM, are necessary to significantly improve electromechanical function.

Pluripotent stem cells, which include ESCs and induced pluripotent stem cells (iPSCs), are promising alternatives owing to their in vitro high proliferative capacity in an undifferentiated state, and their capacity to differentiate into a variety of cell lineages, including CMs. These features allow generation of a great number of cells portraying immature CMs at the phenotypic and functional level. ESC-derived CMs (ESC-CM) display immature contractile machinery and are capable of spontaneous AP generation. This inherent automaticity increases the possibility of induced arrhythmias upon intra-myocardial ESC-CM delivery. Despite that transplantation of pluripotent stem cells-derived CM promote successful engraftment and functional improvement,^29, 30, 83–85^ few studies have thoroughly evaluated their electrically integration and/or pro-arrhythmic properties.^29, 30, 83^ Shiba et al.^29, 83^ showed that, in a guinea pig MI model, human ESC-CM formed cell grafts with calcium transients completely coordinated with ambulatory ECG signals, with evidences of an anti-arrhythmic effect. The same group applied a similar strategy in a non-human primate model of MI showing functional improvement, revascularization, and calcium transients coordinated with ECG signals. Despite these encouraging results, all transplanted animals suffered premature ventricular contractions and ventricular tachycardia.^30^ These opposing results could be explained by the disparity in the heart rate of guinea pigs and macaques. High heart rates, typical of small animal models, could mask the ESC-derived CM automaticity by surpassing their AP generation frequency. In contrast, in large animal models and humans, the automaticity of these cells can be revealed due to slower basal heart rates.^30^ Thus, although these cells show a phenotype which closely mimics CM, their immature state can hinder its clinical applicability. Noteworthy, pluripotent stem cells have other hurdles to surpass such as immunogenicity (especially regarding ESCs), tumorigenic potential due to teratoma formation (although this effect is debatable in the context of the application of ESC-CM^86–88^) or use of viral vectors (mainly in iPSCs), low cell survival and ethical concerns.

In conclusion, an ideal cell capable of adequate cardiac electrical integrity, while improving cardiac function and tissue remodeling, is yet to be described. Regardless, one must reflect on novel therapies on different perspectives: (1) does a certain cell allow sufficient improvement in cardiac function and tissue remodeling that compensates for their pro-arrhythmic potential upon transplantation?; and (2) should we focus on cellular genetic manipulation to approximate their function and phenotype to native CM?

#### Limitations and future directions

One of the most widely recognized issues in cell therapy is the low rate of retention and viability upon transplantation. In particular, this is a relevant problem in MSC delivery to the myocardium.^55, 89^ However, as discussed above, despite their low engraftment, these cells promote functional improvement through the production of paracrine factors.^55, 89^ Thus, very pertinent questions can be raised, should we acknowledge transient cell persistence and subsequent beneficial acute effects, or otherwise invest on improving their engraftment? Do MSC-secreted conditioned medium or isolated factors (e.g., growth factors, exosomes), which have recently shown promising results,^90, 91^ constitute an alternative to cell delivery?

For retention and/or survival improvement, we can either manipulate the host tissue (potentially the least translational option) or subject cells to pre-conditioning before transplantation. Pre-conditioning can involve, incubation of the cells with certain cytokines and/or growth factors,^92^ culture in hypoxic conditions,^93^ genetic modifications^94^ and seeding on biomaterial-based scaffolds that, ideally, would closely resemble the host tissue.^95^ However, following all the efforts to increase cell survival, intrinsic functional and phenotypical differences of the injected cells can result in complications, being arrhythmias caused by heterocellular coupling of MSCs with CMs a good example. Regarding cell sources, the ones that currently are amenable to have a phenotype and function that better matches the myocardium are pluripotent stem cells-derived CMs. However, as discussed in this review, even these cells show a certain degree of immaturity that, can cause deleterious effects, such as arrhythmias resultant from spontaneous beating of ESC-derived CMs when implanted on the myocardium of non-human primates. This suggests that novel strategies to further improve the maturation of these cells are crucial.

To successfully apply cell-based strategies in the clinic setting, one must overcome several technical obstacles. For instance, it is highly recommended that the cell-culture strategy provides adequate cell number for therapy. Also cell-based products must be *off-the-shelf*, which has been successfully developed in the case of MSCs, but is still challenging when considering iPSCs-derived CMs, as derivation of these cells from somatic patient cells still requires extended protocols (~ 4 months). Moreover, it must be proved that the cellular product is consistently safe (e.g., they cannot be tumorigenic) and highly invasive delivery methods should be avoided.

Finally, all cell therapies that reach the clinical setting must be in accordance to ethical issues and general social acceptance. For instance, while ESCs are a controversial source, since these cells are isolated from human embryos, iPSCs rise less ethical questions as they are derived from somatic cells.

## Potential of electrical cues to improve cardiac tissue engineering strategies

Cardiac tissue engineering strategically combines cells, scaffolds and signaling factors to restore cardiac function and/or improve cardiac repair, which can be achieved through different approaches, such as: (1) improving therapies based on cell injection by providing a vehicle to cells and (2) allowing the formation of cardiac tissue constructs in vitro for subsequent implantation (reviewed elsewhere^4, 96^). Although cardiac tissue engineering resulted in promising results, particularly in cell survival and engraftment in vivo, some limitations still remain. For instance, cardiac tissue constructs often exhibit spontaneous contractions (mainly with pluripotent stem cells) which, as already discussed, can have pro-arrhythmic effects. In order to solve this issue, culture conditions have been successively improved by providing biochemical, mechanical, and electrical cues that closely mimic the native myocardial microenvironment.

The role of external electrical stimulation has been tested in vitro on cultured cardiac cells.^97–99^ For instance, Radisic et al.^97^ conducted pioneer work showing that, upon 8 days of in vitro electrical stimulation, cultured neonatal CM exhibited increased alignment, intercellular coupling, ultrastructural organization and amplitude of synchronous contractions, concomitant with improved contractile and electrophysiological proficiency.

Scaffolds containing conductive components have been explored to originate constructs that mimic the myocardial environment, supporting functional cardiac cells and even their electromechanical integration upon transplantation, and/or that promote electrical integrity of the heart by acting directly on native CM. The most explored materials are: (i) gold-based materials, such as gold nanowires (AuNWs) and gold nanoparticles (AuNPs), which exhibit great biocompatibility, low toxicity and, importantly, high electrical conductivity; (ii) carbon-based materials, mainly carbon nanotubes (CNTs) due to their high surface area, high chemical stability, high mechanical strength, and conductivity; (iii) intrinsically electroconductive polymers; and, more recently, (iv) silicon-based approaches. Table 1 summarizes representative studies, some of which will be discussed.

**Table 1:** Experimental results on the application of conductive materials in cardiac tissue engineering.

Material	References	Scaffold	Elastic modulus (kPa)/conductivity (S/m)	Cell source	Main results
Gold	[100]	AuNW-incorporated alginate scaffolds	~ 3.5/n.a.	Neonatal CM	Thick and aligned cell constructs; ↑ α-SA and Cx43; synchronous contractions
	[117]	AuNP-deposited PCL fibers	~ 60 × 10^3^/n.a.	Neonatal CM	Elongated CM, aligned and striated cell constructs; ↑ contraction rate and force
	[103]	AuNP-incorporated biodegradable PU scaffolds	~ 200–240/n.a.	H9C2	Improved cell spreading and alignment; ↑ Nkx2.5, ANF, NPPB expression
	[118]	AuNP-deposited PCL/gelatin scaffolds	n.a./n.a.	Neonatal CM	Elongated CM, visible striation and ↑ aspect ratio; ↑ contraction amplitudes and rates
	[101]	AuNP-deposited decellularized pig omental matrices	~ 12.5 × 10^3^/n.a.	Neonatal CM	Elongated CM, aligned and striated cell constructs; Cx43 between adjacent CM; ↑ contraction amplitude, calcium transient propagation velocity; ↓ excitation threshold
	[119]	AuNP-deposited thiol-HEMA/HEMA scaffolds	~ 600–1600/~ 11–15	Neonatal CM	CM presented as clusters or single cells; 2-fold ↑ Cx43 protein levels
	[120]	AuNW-incorporated GelMA hydrogels	~ 1.3/ n.a.	Neonatal CM	↑ Cell retention and viability; highly organized sarcomeric structures; ↑ beating frequency; more synchronous, stable, and robust beating behavior; synchronized calcium transients; ↓ excitation threshold
Carbon	[106]	MWCNT-embedded PG nanofibers	~ 373.5/n.a.	Neonatal CM	↑ CM alignment, metabolic activity and viability; ↑ Cx43 staining
	[104, 105]	MWCNT solution coating a glass substrate	n.a./n.a.	Neonatal CM	↑ Metabolic activity; more negative membrane resting potential; ↑ αMHC, SERCA2a, Cx43; ↓ ANF
	[121]	Chitosan:CNTs composite scaffolds	~ 28.1/~ 0.25 (hydrated)	Neonatal CM	↑ CM alignment and metabolic activity; ↑ TnI, SERCA2a, GATA4, αMHC, Cx43, βMHC, and ANF expression
	[107]	SWCNT-incorporated gelatin-chitosan hydrogels	~ 19.3 (175 ppm)/n.a.	Neonatal CM	Concentration-dependent cytotoxicity; more developed sarcomeres; ↑ α-SA; intercellular Cx43 staining; ↑ beating rates and conduction velocity; ↓ AP duration
	[122]	CNT-embedded GelMA hydrogels	~ 20–54/n.a.	Neonatal CM	Aligned, interconnected CM; developed sarcomeres; attenuation of heptanol-induced intercellular coupling inhibition
	[123]	PLGA:CNFs composite substrates	n.a./~ 5 × 10^−4^ – 7 × 10^−3^	Human CM, rat EC, NIH/3T3	↑ CM density; ↓ ECs and fibroblast growth
	[109]	SWCNT/collagen solution coating a glass substrate	n.a./~ 1.90 × 10^−8^–1.77 × 10^3^	Neonatal CM	Marked striation and organized sarcomeres; functional beating syncytium; ↑TnI, Cx43, N-cadherin, plakophilin2 and plakoglobin expression; well-developed intercalated disc junctions; ↑ β1-integrin, FAK, p-ERK, MEF-2c and GATA4
	[108]	SWCNT-incorporated gelatin hydrogels	n.a. (shear modulus:~ 20–400 Pa)/ ~ 5 × 10^−5^	Neonatal CM	In vitro: aligned cell constructs; organized sarcomeres;↑ α-SA and Cx43 levels; spontaneous electrical activity;
					In vivo (MI rats)*:*↑ Cx43, Na_V_1.5, and N-cadherin protein levels; unclear scaffold/scar boundary; presence of smooth-muscle cells and CD68^+^ macrophages;↑ ejection fraction and fractional shortening. Electrical coupling was assessed by evaluating Cx43
Conductive polymers	[111]	PCL/PU blend scaffolds containing aniline pentamers	~ 1.3 × 10^3^/~ 10^−4^–10^4^	Neonatal CM	↑ TnT, Cx43, actinin-α-4
	[110]	Nanofibrous 2D meshes of HCl-doped PANI/PLGA blend	~ 91.7 × 10^3^/~ 0.31	Neonatal CM	Isolated cell clusters; spontaneous beating activity; ↑ TnI, Cx43 expression; intercellular Cx43 localization
	[112]	PPy/PCL/gelatin blend nanofibers on glass substrate	~ 16.8 × 10^3^/~ 1.3 × 10^−3^	Rabbit CM	↑ α-SA, TnT and Cx43; increasing PPy proportion disrupted mechanical properties and slowed CM growth
	[114]	Injectable PPy-grafted chitosan hydrogel	~ 2/~ 0.02	Neonatal CM	In vitro:↑ Ca^2+^ transients velocity;
					In vivo (MI rats)*:*QRS interval duration similar to healthy;↑ transverse and border zone/scar region conduction velocities;↑ ejection fraction, d*P*/d*t* max and min, preload recruitable stroke work;
					Note: although ex vivo optical mapping was performed to assess conduction velocities at the injection site, since the hydrogel was injected without cells, electrical coupling between implanted cells and the native myocardium was not evaluated.
	[113]	Films of interpenetrating PPy and PCL networks	~ 9.3 × 10^5^/~ 0.10	HL-1	↑ Proportion of cells with peripheral Cx43 expression;↑ Ca^2+^ transients velocity and spontaneous electrical activity frequency
	[115]	Films of chitosan and PANI	~ 6.7 × 10^3^/~ 16	n.a.	Ex vivo: rat cardiac slices - ↓ transverse and longitudinal conduction velocities; Whole rat hearts (optical mapping) - ↓ conduction velocities
					In vivo (healthy rats): unaffected ejection fraction, fractional shortening and no aggravated arrhythmia inducibility
Silicon	[116]	SiNW-incorporated cardiac cell spheroids	n.a./150–500	Neonatal CMs or hiPSC-derived CMs	Improved intercellular coupling (e.g., ↑ Cx43 and N-cadherin); improved contractile machinery development; ↑ β-MHC/α-MHC ratio; ↓ spontaneous beating frequency

*n.a*. non-available, *αMHC* alpha myosin heavy chain, *α-SA* alpha-sarcomericactinin, *βMHC* beta myosin heavy chain. *ANF* atrial natriuretic factor, *AuNP* gold nanoparticle, *AuNW* gold nanowire, *CM* cardiomyocyte, *CNT* carbon nanotube, *CNF* carbon nanofibers, *Cx43* connexin-43, *EC* endothelial cell, *ERK* extracellular-signal-regulated kinase, *ESC* embryonic stem cell, *FAK* focal adhesion kinase, *GelMA* gelatin methacrylate, *HEMA* hydroxyethylmethacrylate, *hiPSCs* human-induced pluripotent stem cells, *MEF-2c* myocyte-specific enhancer factor 2C, *MHC* myosin heavy chain, *MI* myocardial infarction, *MWCNT* multi-walled carbon nanotube, *NPPB* natriuretic peptide precursor B, *PANI* polyaniline, *PCL* polycaprolactone, *PECAM1* platelet endothelial cell adhesion molecule 1, *PLGA* polylactic-co-glycolic acid, *PPy* polypyrrole, *PU* polyurethane, *SERCA2a* sarcoplasmic reticulum Ca^2+^ ATPase 2a, *SiNW* silicon nanowire, *SWCNT* single-walled carbon nanotube, *TnI* troponin I, *TnT* troponin

### Gold-based materials

In a pioneer study, Dvir et al.^100^ integrated AuNWs in the pore walls of macroporous alginate scaffolds. Neonatal CMs were cultured on AuNWs scaffolds during 3 days without electrical stimulation followed by 5 days of electrical stimulation. While alginate-only scaffolds resulted in the formation of small cell aggregates within the pores, Au-NW scaffolds showed thick and aligned cardiac cell constructs. Furthermore, CMs in these scaffolds exhibited Ca^2+^ transients, synchronous contraction and higher content of α-sarcomeric actinin and Cx43. Aiming to mimic the native cardiac ECM fibrous organization, AuNPs deposited on fibrous decellularized pig omental matrices promoted Cx43 rearrangement between adjacent neonatal CM organized in elongated, aligned, and striated cell constructs with stronger contraction forces.^101^ To assure proper alignment and electrical coupling a cardiac patch should also be mechanically compatible with the myocardium. Myocardial stiffness in humans is approximately 10 kPa and 500 kPa at the beginning and end of diastole, respectively.^102^ Of note, the majority of studies with gold-based materials summarized in Table 1 report scaffold’s stiffness out of this range. Recently, this issue has been addressed through incorporation of AuNWs into biodegradable polyurethane porous scaffolds with an elasticity of 200 to 240 kPa. Electrical stimulation improved H9C2 rat CM spreading and alignment, however, Cx43 expression was unaffected.^103^

Although the aim of the aforementioned studies was the design a conductive gold-based scaffold that would closely mimic the myocardial tissue, to our best knowledge, the applicability or therapeutic evaluation of these strategies in vivo, is yet to be performed.

### Carbon-based materials

Initial studies showed that neonatal CMs cultured in vitro on the top of CNTs on glass substrates presented higher metabolic activity and proliferation rates, and displayed larger domains of syncytial beating. Importantly, whole-cell patch clamp recording showed that CMs cultured on CNTs for 3 days exhibited more negative membrane resting potential than controls, evidencing increased CM maturation.^104^ The improved maturation of CMs on CNTs was attributed to more abundant Cx43 gap junctions and alterations on gene expression.^105^

These in vitro 2D approaches evolved to the use of carbon-based three-dimensional ECM-like scaffolds such as scaffolds comprising poly (glycerol sebacate):gelatin aligned electrospun nanofiber embedded with CNTs. Neonatal CMs cultured on CNT-containing scaffolds had higher viability, metabolic activity, increased Cx43 levels, became more aligned and displayed higher beating rates and lower excitation threshold than CNT-free counterparts.^106^ An independent study showed that CM cultured on CNT scaffolds augmented conduction velocity, reduced AP duration, and exhibited preferential localization of Cx43 at cell–cell junctions. Of note, potential cytotoxic effects of CNTs were demonstrated at high concentrations (175 ppm) (ref. 107). In 2014, CNT-based composite cardiac patches were for the first time tested in vivo. CNT-gelatin scaffolds seeded with neonatal CM were delivered intramyocardially to rats 14 days after MI. Following 4 weeks of implantation, the boundary between the scaffold and scar tissue was unclear and CNT-seeded CM presented upregulated levels of Cx43, Na_V_1.5 and N-cadherin (typical of intercalated discs). Interestingly, a portion of CNT-seeded CM migrated to the scar tissue, along with CNTs. The scaffold also contained host-derived cells such as CM, smooth muscle cells, and CD68^+^ macrophages, showing evidences that the scaffold integrated with the host cardiac tissue. Heart function assessment showed that ejection fraction and fractional shortening were significantly improved when compared to control groups (i.e., sham, scaffolds without CNTs and scaffolds seeded with cardiac fibroblasts).^108^ However, the mentioned groups were not compared with the injection of CMs without scaffold, which could be useful to determine if the biomaterial is not only relevant in improving cardiac function, but also in providing an improvement in the engraftment ability and coupling of the cells with the native tissue; nor were compared with conductive scaffolds without cells. Despite the aforementioned beneficial effect of CM in CNTs, electromechanical coupling with native counterparts and electrophysiological impact is yet to be demonstrated.

Although the majority of the described studies show improved intercellular electrical coupling induced by carbon-based materials, the molecular mechanisms that trigger these effects are still not well understood. A recent report further demonstrated that CNT-collagen seeded CM-induced intercalated disc gap junctions assembly via β1-integrin/FAK/ERK/MEF-2c and GATA4 signaling pathway.^109^ Of note, it remained unclear if CNT-collagen observed effects could be attributed to mechanical and/or to electrical cues.

### Conductive polymers-based materials

The two main studied polymers are polyaniline (PANI) and polypyrrole (PPy). Some authors explored in vitro the capability of PANI-based materials to promote functional proficiency and electrical coupling of CM.^110, 111^ Baheiraei and co-workers explored the properties of porous scaffolds composed of polycaprolactone and a biodegradable polyurethane polymer containing aniline pentamers. This strategy aimed to harness the electroconductive properties of the aniline pentamers while trying to surpass the low biodegradability and poor mechanical properties of PANI. Conductive scaffolds promoted neonatal CM adhesion, growth and higher expression of genes involved in contraction and cytoskeleton alignment, however; Cx43 expression was not significantly altered.^111^

Regarding PPy-based materials, CM were cultured in vitro on electrospun nanofibers consisting in blend of doped PPy, polycaprolactone and gelatin onto glass coverslips. Overall, increasing the concentration of PPy (0–30%) increased tensile modulus: 15% PPy presented better conductivity, mechanical properties, and biodegradability with seeded CM displaying enhanced performance considering cell attachment, proliferation, interaction, and expression of cardiac-specific proteins.^112^ More recently, Spearman et al.^113^ produced films comprising interpenetrating networks of PPy and polycaprolactone. HL-1 atrial myocytes seeded on these films remained viable, became more elongated, presented higher levels of Cx43 and increased calcium transient propagation velocity and spontaneous electrical activity frequency values.

Similarly to the other conductive materials, in vivo studies involving electroconductive polymers are scarce and dedicated to cell-free injectable PPy-based hydrogels rather than to tissue engineered cardiac patches.^114^ Notwithstanding, a recent study addressed the effect of an injectable hydrogel composed of PPy-grafted chitosan, on cardiac function and AP propagation in a rat MI model. The hydrogel was administered to the infarcted LV 1 week after MI. Eight weeks after injection the treated group showed improved LV function and QRS interval duration of PPy/chitosan-injected heart was similar to that of healthy animals. Furthermore, ex vivo optical mapping showed that the conduction velocities at the border zone/scar region were significantly increased in the PPy/chitosan-injected hearts.^114^ Of note, these in vivo experiments involved the injection of the polymer alone. Considering that the biomaterial also improved electrical coupling and calcium handling of the neonatal CMs in vitro, it would be interesting to assess whether these injectable polymers enhance engraftment (mechanical and/or electrical) of implanted cells. This would also clarify whether adding CM to the scaffolds prior to implantation would synergize and provide an additional functional improvement.^114^ More recently, Molly Stevens’ group designed conductive films composed of chitosan and PANI. Ex vivo optical mapping performed on whole rat hearts (both healthy or with MI) showed that patches reduced cardiac conduction velocities when applied on the epicardium. Thus, although patches exerted a negative effect on cardiac electrical integrity, they were successful in altering the electrophysiological properties of the tissue. In addition, patch implantation on the epicardium of healthy hearts showed no negative effects on echocardiographic and electrocardiographic parameters, 2 weeks after implantation.^115^ Of note, in this study, the effect of the films on cultured cells was not evaluated (neither in vitro nor in vivo) and long-term functional assessments in patch-implanted animals (either in physiological or pathological conditions) were lacking.

### Silicon-based approaches

Recently, when incorporated in spheroids comprising neonatal rat CM or human-induced pluripotent stem cells (hiPSCs)-derived CM, silicon nanowires (SiNWs) were shown to enhance contractility and synchronization and to increase expression of α-sarcomeric actinin and Cx43 (ref. 116). Furthermore, SiNWs and electrical stimulation had a synergistic effect on hiPSC-derived CM spheroids which presented more developed sarcomeric apparatus, improved intercellular coupling, higher degree of maturation and reduced spontaneous beating frequency.^116^

### Limitations and future directions

Overall, scaffolds based on conductive materials showed promising results as they potentiate the formation of aligned, striated, and synchronously contracting CMs, exhibiting increased calcium transients and AP propagation velocities and higher contractility forces (Table 1). Additionally, upregulation of genes associated with CM maturation and/or functional proficiency was also observed (Table 1). In common with other cardiac tissue engineering approaches, constructs were often beating spontaneously indicating that electrical cues were not sufficient to obtain full maturation, although it is still to verify whether full maturation would be obtained following implantation. In line with this, some authors appear to consider an increase in beating frequency as a positive outcome, however, it could be associated with increased automaticity and, thus, a pro-arrhythmic tendency upon implantation. It should also be noted the scarcity of in vivo studies involving conductive materials, which in general lack systematic evaluation to answer pertinent questions such as: (1) Do conductive materials exert a beneficial effect by themselves, or pre-seeding of scaffolds with cells greatly improves the outcome? (2) Do the biomaterials improve cardiac electrical integrity by restoring the electrical activity of the host myocardial tissue in an indirect manner and/or by establishing electrical coupling between implanted cells and surrounding tissue? (3) Do implanted biomaterials support AP propagation through their structure? (4) What are the molecular mechanisms underlying improvement on electrical integrity? Importantly, to ascertain whether these materials have clinical applicability in a near-future, in vivo studies in larger animal models (e.g., pig, that exhibit an electrophysiological behavior closer to humans) associated with thorough electrophysiological assessments are required. Apart from their potential for altering in vivo electrical integrity, other essential issues such as biocompatibility, concentration-dependent toxicity (especially for CNTs) and poor mechanical properties (mainly for electroconductive polymers) should also be evaluated.

## Final remarks

The reported progresses clearly show the relevance of considering cardiac electrical integrity as a central objective of innovative bioengineered approaches. Promising results stemmed from both gene and cell therapies whose main goal comprised acting directly on restoring a defective electrical conduction system. These results further underlined that, for instance, in a CVD setting involving extensive loss of CM, as is the case of MI, an efficient therapy must rely not only in replacing CM but also in restoring myocardial electrical integrity and/or arrhythmogenicity. In that line, conductive biomaterials coupled or not with external electrical stimulation enhanced the outcome of conventional tissue engineering strategies by promoting cells/biomaterial/native myocardium electromechanical integration.

**Fig. 3:**
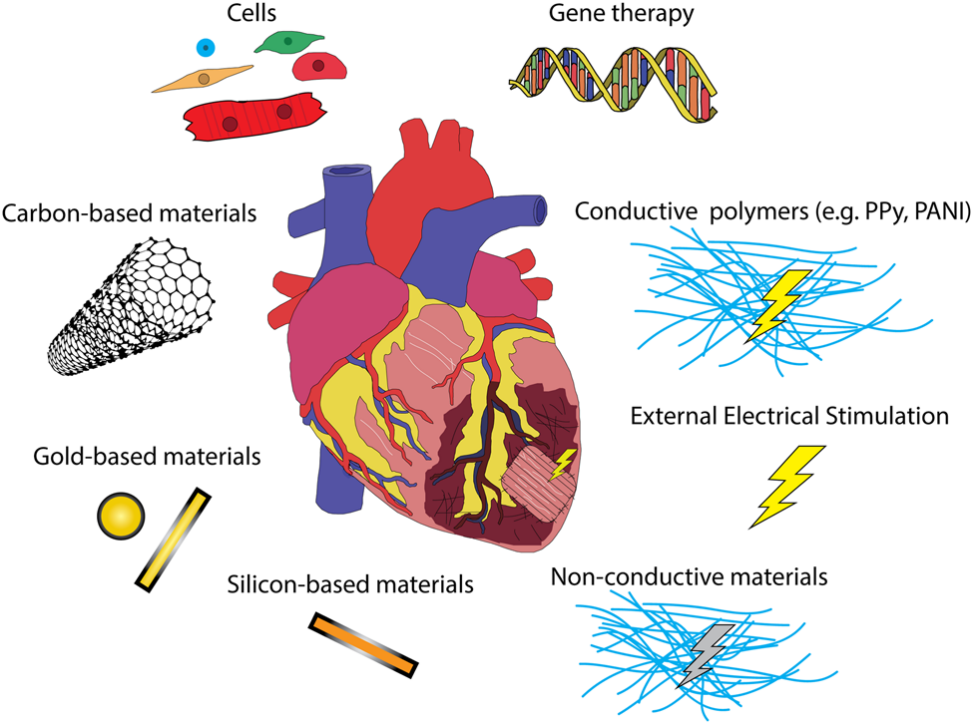
Summary of discussed approaches with great potential to restore electrical integrity, and that are amenable to be combined as an electromechanical integrated biomaterial-based patch

Future studies exploring combinatory therapies to promote electrical integrity and CM replacement in clinically relevant in vivo models and further dissection of the underlying cellular and molecular mechanisms, are warranted (Fig. 3). In order to optimize combinatorial strategies, one must apply *state-of-art* knowledge concerning the shortcomings and assets of each approach, i.e., gene therapy, cell therapy, and tissue engineering (that includes biomaterials, soluble factors, cell constructs, and bioreactors). In that sense, one can reflect on how these elements can synergistically promote functional improvement in a disease scenario. For example, it could be envisaged a conductive scaffold for MSC delivery that would improve myocardial electrical activity and survival of MSC, while allowing the diffusion of secreted factors to the surrounding tissue. Conversely, when aiming the use of cardiac constructs with iPSC-derived CMs, a great effort should be directed to produce mature CMs and avoid complications such as automaticity-induced arrhythmogenesis. In that sense, factors or vectors, for gene/mRNA delivery, could be integrated in conductive scaffolds to potentiate the differentiation of pluripotent stem cells to mature CMs. In addition, it would be recommended the use of a biodegradable scaffold (e.g., chitosan) so that, in the end, only CM engrafted in the native myocardium, although the conductive properties could be useful in an initial phase to promote the intercellular electrical coupling of the construct with the neighboring host cells.

## References

[1] Mozaffarian D (2016). Heart disease and stroke statistics-2016 update: A report From the American heart association. Circulation.

[2] Kleber AG, Rudy Y (2004). Basic mechanisms of cardiac impulse propagation and associated arrhythmias. Physiol. Rev..

[3] Lip GY (2015). European heart rhythm association/heart failure association joint consensus document on arrhythmias in heart failure, endorsed by the Heart rhythm society and the Asia Pacific heart rhythm society. Eur.J. Heart Fail..

[4] Hirt MN, Hansen A, Eschenhagen T (2014). Cardiac tissue engineering: state of the art. Circ. Res..

[5] Motloch LJ, Akar FG (2015). Gene therapy to restore electrophysiological function in heart failure. Expert Opin. Biol. Ther..

[6] Shimada T, Kawazato H, Yasuda A, Ono N, Sueda K (2004). Cytoarchitecture and intercalated disks of the working myocardium and the conduction system in the mammalian heart. Anat. Rec. A. Discov. Mol. Cell. Evol. Biol..

[7] Blank AC (2009). Rewiring the heart: stem cell therapy to restore normal cardiac excitability and conduction. Curr. Stem Cell Res. Ther..

[8] Veerman CC, Wilde AA, Lodder EM (2015). The cardiac sodium channel gene SCN5A and its gene product NaV1.5: Role in physiology and pathophysiology. Gene.

[9] Thiriet, M. in *Tissue Functioning and Remodeling in the Circulatory and Ventilatory Systems* 1st edn, Vol. 5 Ch. 5 (Springer-Verlag, 2013).

[10] Amin AS, Tan HL, Wilde AA (2010). Cardiac ion channels in health and disease. Heart Rhythm.

[11] Weiss S, Oz S, Benmocha A, Dascal N (2013). Regulation of cardiac L-Type Ca2+channel CaV1.2 Via the β-Adrenergic-cAMP-protein kinase a pathway: Old dogmas, advances, and New uncertainties. Circ. Res..

[12] Robinson RB, Siegelbaum SA (2003). Hyperpolarization-activated cation currents: from molecules to physiological function. Annu. Rev. Physiol..

[13] Lakatta EG, Maltsev VA, Vinogradova TM (2010). A coupled SYSTEM of intracellular Ca2+clocks and surface membrane voltage clocks controls the timekeeping mechanism of the heart’s pacemaker. Circ. Res..

[14] Jansen JA, van Veen TAB, de Bakker JMT, van Rijen HVM (2010). Cardiac connexins and impulse propagation. J. Mol. Cell. Cardiol..

[15] Davis LM, Kanter HL, Beyer EC, Saffitz JE (1994). Distinct gap junction protein phenotypes in cardiac tissues with disparate conduction properties. J. Am. Coll. Cardiol..

[16] Severs NJ, Bruce AF, Dupont E, Rothery S (2008). Remodelling of gap junctions and connexin expression in diseased myocardium. Cardiovasc. Res..

[17] Gourdie RG (1993). The spatial distribution and relative abundance of gap-junctional connexin40 and connexin43 correlate to functional properties of components of the cardiac atrioventricular conduction system. J. Cell Sci..

[18] Kanter HL, Laing JG, Beau SL, Beyer EC, Saffitz JE (1993). Distinct patterns of connexin expression in canine Purkinje fibers and ventricular muscle. Circ. Res..

[19] Temple IP, Inada S, Dobrzynski H, Boyett MR (2013). Connexins and the atrioventricular node. Heart Rhythm.

[20] Veenstra RD, Wang HZ, Westphale EM, Beyer EC (1992). Multiple connexins confer distinct regulatory and conductance properties of gap junctions in developing heart. Circ. Res..

[21] Davis LM, Rodefeld ME, Green K, Beyer EC, Saffitz JE (1995). Gap junction protein phenotypes of the human heart and conduction system. J. Cardiovasc. Electrophysiol..

[22] Veeraraghavan R, Poelzing S, Gourdie RG (2014). Intercellular electrical communication in the heart: a new, active role for the intercalated disk. Cell Commun. Adhes..

[23] Coronel R (2013). Electrophysiological changes in heart failure and their implications for arrhythmogenesis. Biochim. Biophys. Acta..

[24] Schmidt U (1998). Contribution of abnormal sarcoplasmic reticulum ATPase activity to systolic and diastolic dysfunction in human heart failure. J. Mol. Cell. Cardiol..

[25] Ai X, Pogwizd SM (2005). Connexin 43 downregulation and dephosphorylation in nonischemic heart failure is associated with enhanced colocalized protein phosphatase type 2A. Circ. Res..

[26] Waldo AL (1996). Effect of d-sotalol on mortality in patients with left ventricular dysfunction after recent and remote myocardial infarction. The SWORD Investigators. Survival with oral d-Sotalol. Lancet (London, England).

[27] Berul CI (2003). Electrophysiological phenotyping in genetically engineered mice. Physiol. Genomics.

[28] Lee P (2012). *In Situ*optical mapping of voltage and calcium in the heart. PLoS ONE..

[29] Shiba Y (2014). Electrical integration of human embryonic stem cell-derived cardiomyocytes in a guinea pig chronic infarct model. J. Cardiovasc. Pharmacol. Ther..

[30] Chong JJ (2014). Human embryonic-stem-cell-derived cardiomyocytes regenerate non-human primate hearts. Nature.

[31] Greener ID (2012). Connexin43 gene transfer reduces ventricular tachycardia susceptibility after myocardial infarction. J. Am. Coll. Cardiol..

[32] Igarashi T (2012). Connexin gene transfer preserves conduction velocity and prevents atrial fibrillation. Circulation.

[33] Boink GJ (2012). SkM1 and Cx32 improve conduction in canine myocardial infarcts yet only SkM1 is antiarrhythmic. Cardiovasc. Res..

[34] Protas L (2009). Expression of skeletal but not cardiac Na(+) channel isoform preserves normal conduction in a depolarized cardiac syncytium. Cardiovasc. Res..

[35] Lau DH (2009). Epicardial border zone overexpression of skeletal muscle sodium channel SkM1 normalizes activation, preserves conduction, and suppresses ventricular arrhythmia: an in silico, in vivo, in vitro study. Circulation.

[36] Coronel R (2010). Cardiac expression of skeletal muscle sodium channels increases longitudinal conduction velocity in the canine 1-week myocardial infarction. Heart Rhythm.

[37] Miyamoto MI (2000). Adenoviral gene transfer of SERCA2a improves left-ventricular function in aortic-banded rats in transition to heart failure. Proc. Natl Acad. Sci. U. S. A..

[38] Lyon AR (2011). SERCA2a gene transfer decreases sarcoplasmic reticulum calcium leak and reduces ventricular arrhythmias in a model of chronic heart failure. Circ. Arrhythm. Electrophysiol..

[39] Cutler MJ (2012). Targeted sarcoplasmic reticulum Ca2+ATPase 2a gene delivery to restore electrical stability in the failing heart. Circulation.

[40] Jaski BE (2009). Calcium upregulation by percutaneous administration of gene therapy in cardiac disease (CUPID Trial), a first-in-human phase 1/2 clinical trial. J. Card. Fail..

[41] Zsebo K (2014). Long-term effects of AAV1/SERCA2a gene transfer in patients with severe heart failure: analysis of recurrent cardiovascular events and mortality. Circ. Res..

[42] Greenberg B (2016). Calcium upregulation by percutaneous administration of gene therapy in patients with cardiac disease (CUPID 2): a randomised, multinational, double-blind, placebo-controlled, phase 2b trial. The Lancet.

[43] Mingozzi F (2013). Overcoming preexisting humoral immunity to AAV using capsid decoys. Sci. Transl. Med..

[44] Prunier F (2008). Prevention of ventricular arrhythmias with sarcoplasmic reticulum Ca2+ATPase pump overexpression in a porcine model of ischemia reperfusion. Circulation.

[45] del Monte F (2004). Abrogation of ventricular arrhythmias in a model of ischemia and reperfusion by targeting myocardial calcium cycling. Proc. Natl Acad. Sci. U.S.A..

[46] Kanno S, Kovacs A, Yamada KA, Saffitz JE (2003). Connexin43 as a determinant of myocardial infarct size following coronary occlusion in mice. J. Am. Coll. Cardiol..

[47] Kaplitt MG (1996). Long-term gene transfer in porcine myocardium after coronary infusion of an adeno-associated virus vector. Ann. Thorac. Surg..

[48] Zincarelli C, Soltys S, Rengo G, Koch WJ, Rabinowitz JE (2010). Comparative cardiac gene delivery of adeno-associated virus serotypes 1-9 reveals that AAV6 mediates the most efficient transduction in mouse heart. Clin. Transl. Sci..

[49] Sander JD, Joung JK (2014). CRISPR-Cas systems for editing, regulating and targeting genomes. Nat. Biotech..

[50] Plotnikov AN (2004). Biological pacemaker implanted in canine left bundle branch provides ventricular escape rhythms that have physiologically acceptable rates. Circulation.

[51] Pacak CA, Sakai Y, Thattaliyath BD, Mah CS, Byrne BJ (2008). Tissue specific promoters improve specificity of AAV9 mediated transgene expression following intra-vascular gene delivery in neonatal mice. Genet. Vaccines Ther..

[52] Sanganalmath SK, Bolli R (2013). Cell therapy for heart failure: a comprehensive overview of experimental and clinical studies, current challenges, and future directions. Circ. Res..

[53] Mathiasen AB (2015). Bone marrow-derived mesenchymal stromal cell treatment in patients with severe ischaemic heart failure: a randomized placebo-controlled trial (MSC-HF trial). Eur. Heart. J..

[54] He J (2013). Injection of Sca-1+/CD45+/CD31+mouse bone mesenchymal stromal-like cells improves cardiac function in a mouse myocardial infarct model. Differentiation.

[55] Nascimento DS (2014). Human umbilical cord tissue-derived mesenchymal stromal cells attenuate remodeling after myocardial infarction by proangiogenic, antiapoptotic, and endogenous cell-activation mechanisms. Stem Cell Res.Ther..

[56] Price MJ (2006). Intravenous mesenchymal stem cell therapy early after reperfused acute myocardial infarction improves left ventricular function and alters electrophysiologic properties. Int. J. Cardiol..

[57] Kim SK (2010). Cardiac cell therapy with mesenchymal stem cell induces cardiac nerve sprouting, angiogenesis, and reduced connexin43-positive gap junctions, but concomitant electrical pacing increases connexin43-positive gap junctions in canine heart. Cardiol. Young.

[58] Chen SL (2004). Improvement of cardiac function after transplantation of autologous bone marrow mesenchymal stem cells in patients with acute myocardial infarction. Chin. Med. J..

[59] Katritsis DG, Sotiropoulou P, Giazitzoglou E, Karvouni E, Papamichail M (2007). Electrophysiological effects of intracoronary transplantation of autologous mesenchymal and endothelial progenitor cells. Europace.

[60] Mureli S (2013). Mesenchymal stem cells improve cardiac conduction by upregulation of connexin 43 through paracrine signaling. Am. J. Physiol. Heart. Circ. Physiol..

[61] Wang D (2011). Mesenchymal stem cell injection ameliorates the inducibility of ventricular arrhythmias after myocardial infarction in rats. Int. J. Cardiol..

[62] Wei F (2012). Mesenchymal stem cells neither fully acquire the electrophysiological properties of mature cardiomyocytes nor promote ventricular arrhythmias in infarcted rats. Basic Res. Cardiol..

[63] Chang MG (2006). Proarrhythmic potential of mesenchymal stem cell transplantation revealed in an in vitro coculture model. Circulation.

[64] Askar SF (2013). Engraftment patterns of human adult mesenchymal stem cells expose electrotonic and paracrine proarrhythmic mechanisms in myocardial cell cultures. Circ. Arrhythm. Electrophysiol..

[65] Mills WR (2007). Stem cell therapy enhances electrical viability in myocardial infarction. J. Mol. Cell. Cardiol..

[66] Pak HN (2003). Mesenchymal stem cell injection induces cardiac nerve sprouting and increased tenascin expression in a Swine model of myocardial infarction. J. Cardiovasc. Electrophysiol..

[67] Pijnappels DA (2006). Progressive increase in conduction velocity across human mesenchymal stem cells is mediated by enhanced electrical coupling. Cardiovasc. Res..

[68] Beeres SLMA (2005). Human adult bone marrow mesenchymal stem cells repair experimental conduction block in rat cardiomyocyte cultures. J. Am. Coll. Cardiol..

[69] Vasquez C, Benamer N, Morley GE (2011). The cardiac fibroblast: functional and electrophysiological considerations in healthy and diseased hearts. J. Cardiovasc. Pharmacol..

[70] Goshima K, Tonomura Y (1969). Synchronized beating of embryonic mouse myocardial cells mediated by FL cells in monolayer culture. Exp. Cell Res..

[71] Yokokawa M (2008). Transplantation of mesenchymal stem cells improves atrioventricular conduction in a rat model of complete atrioventricular block. Cell Transplant..

[72] Durrani S, Konoplyannikov M, Ashraf M, Haider KH (2010). Skeletal myoblasts for cardiac repair. Regen. Med..

[73] Menasche P (2008). The myoblast autologous grafting in ischemic cardiomyopathy (MAGIC) trial: first randomized placebo-controlled study of myoblast transplantation. Circulation.

[74] Fernandes S (2006). Autologous myoblast transplantation after myocardial infarction increases the inducibility of ventricular arrhythmias. Cardiovasc. Res..

[75] Suzuki K (2001). Overexpression of connexin 43 in skeletal myoblasts: Relevance to cell transplantation to the heart. J. Thorac. Cardiovasc. Surg..

[76] Tolmachov O (2006). Overexpression of connexin 43 using a retroviral vector improves electrical coupling of skeletal myoblasts with cardiac myocytes in vitro. BMC Cardiovasc. Disord..

[77] Choi YH (2006). Cardiac conduction through engineered tissue. Am. J. Pathol..

[78] Roell W (2007). Engraftment of connexin 43-expressing cells prevents post-infarct arrhythmia. Nature.

[79] Antanaviciute I (2015). Exogenous connexin43-expressing autologous skeletal myoblasts ameliorate mechanical function and electrical activity of the rabbit heart after experimental infarction. Int. J. Exp. Pathol..

[80] Kolanowski TJ (2014). In vitro and in vivo characteristics of connexin 43-modified human skeletal myoblasts as candidates for prospective stem cell therapy for the failing heart. Int. J. Cardiol..

[81] Fernandes S (2009). Cardiac cell therapy: overexpression of connexin43 in skeletal myoblasts and prevention of ventricular arrhythmias. J. Cell. Mol. Med..

[82] Ott HC (2004). On the fate of skeletal myoblasts in a cardiac environment: down-regulation of voltage-gated ion channels. J. Physiol..

[83] Shiba Y (2012). Human ES-cell-derived cardiomyocytes electrically couple and suppress arrhythmias in injured hearts. Nature.

[84] Ye J (2015). Treatment with hESC-derived myocardial precursors improves cardiac function after a myocardial infarction. PLoS ONE.

[85] Mauritz C (2011). Induced pluripotent stem cell (iPSC)-derived Flk-1 progenitor cells engraft, differentiate, and improve heart function in a mouse model of acute myocardial infarction. Eur. Heart J..

[86] Hattori F, Fukuda K (2010). Strategies for ensuring that regenerative cardiomyocytes function properly and in cooperation with the host myocardium. Exp. Mol. Med..

[87] Kawamura A (2016). Teratocarcinomas arising from allogeneic induced pluripotent stem cell-derived cardiac tissue constructs provoked host immune rejection in mice. Sci.Rep..

[88] Liu Z (2013). The tumourigenicity of iPS cells and their differentiated derivates. J. Cell. Mol. Med..

[89] Cai M (2016). Bone marrow mesenchymal stem cells (BM-MSCs) improve heart function in swine myocardial infarction model through paracrine effects. Sci. Rep..

[90] Timmers L (2011). Human mesenchymal stem cell-conditioned medium improves cardiac function following myocardial infarction. Stem Cell Res..

[91] Arslan F (2013). Mesenchymal stem cell-derived exosomes increase ATP levels, decrease oxidative stress and activate PI3K/Akt pathway to enhance myocardial viability and prevent adverse remodeling after myocardial ischemia/reperfusion injury. Stem Cell Res..

[92] Herrmann JL (2010). Preconditioning mesenchymal stem cells with transforming growth factor-alpha improves mesenchymal stem cell-mediated cardioprotection. Shock (Augusta, Ga).

[93] Hu X (2008). Transplantation of hypoxia-preconditioned mesenchymal stem cells improves infarcted heart function via enhanced survival of implanted cells and angiogenesis. J. Thorac. Cardiovasc. Surg..

[94] Li W (2007). Bcl-2 engineered MSCs inhibited apoptosis and improved heart function. Stem Cells.

[95] Li L, Chen X, Wang WE, Zeng C (2016). How to improve the survival of transplanted mesenchymal stem cell in ischemic heart?. Stem Cells Int..

[96] Reis LA, Chiu LL, Feric N, Fu L, Radisic M (2016). Biomaterials in myocardial tissue engineering. J. Tissue Eng. Regen. Med..

[97] Radisic M (2004). Functional assembly of engineered myocardium by electrical stimulation of cardiac myocytes cultured on scaffolds. Proc. Natl Acad. Sci. U. S. A..

[98] Au HT, Cheng I, Chowdhury MF, Radisic M (2007). Interactive effects of surface topography and pulsatile electrical field stimulation on orientation and elongation of fibroblasts and cardiomyocytes. Biomaterials.

[99] Miklas JW (2014). Bioreactor for modulation of cardiac microtissue phenotype by combined static stretch and electrical stimulation. Biofabrication.

[100] Dvir T (2011). Nanowired three-dimensional cardiac patches. Nat. Nanotechnol..

[101] Shevach M, Fleischer S, Shapira A, Dvir T (2014). Gold nanoparticle-decellularized matrix hybrids for cardiac tissue engineering. Nano. Lett..

[102] Chen Q-Z (2008). Characterisation of a soft elastomer poly(glycerol sebacate) designed to match the mechanical properties of myocardial tissue. Biomaterials.

[103] Ganji Y (2016). Cardiomyocyte behavior on biodegradable polyurethane/gold nanocomposite scaffolds under electrical stimulation. Mater. Sci. Eng.: C.

[104] Martinelli V (2012). Carbon nanotubes promote growth and spontaneous electrical activity in cultured cardiac myocytes. Nano. Lett..

[105] Martinelli V (2013). Carbon nanotubes instruct physiological growth and functionally mature syncytia: Nongenetic engineering of cardiac myocytes. ACS Nano.

[106] Kharaziha M (2014). Tough and flexible CNT-polymeric hybrid scaffolds for engineering cardiac constructs. Biomaterials.

[107] Pok S (2014). Biocompatible carbon nanotube–chitosan scaffold matching the electrical conductivity of the heart. ACS Nano.

[108] Zhou J (2014). Engineering the heart: evaluation of conductive nanomaterials for improving implant integration and cardiac function. Sci. Rep..

[109] Sun H (2015). Carbon nanotubes enhance intercalated disc assembly in cardiac myocytes via the beta1-integrin-mediated signaling pathway. Biomaterials.

[110] Hsiao CW (2013). Electrical coupling of isolated cardiomyocyte clusters grown on aligned conductive nanofibrous meshes for their synchronized beating. Biomaterials.

[111] Baheiraei N (2015). Preparation of a porous conductive scaffold from aniline pentamer-modified polyurethane/PCL blend for cardiac tissue engineering. J. Biomed. Mater. Res. A..

[112] Kai D, Prabhakaran MP, Jin G, Ramakrishna S (2011). Polypyrrole-contained electrospun conductive nanofibrous membranes for cardiac tissue engineering. J. Biomed. Mater. Res.A..

[113] Spearman BS (2015). Conductive interpenetrating networks of polypyrrole and polycaprolactone encourage electrophysiological development of cardiac cells. Acta Biomater..

[114] Mihic A (2015). A conductive polymer hydrogel supports cell electrical signaling and improves cardiac function after implantation into myocardial infarct. Circulation.

[115] Mawad D (2016). A conducting polymer with enhanced electronic stability applied in cardiac models. Sci. Adv..

[116] Richards DJ (2016). Nanowires and electrical stimulation synergistically improve functions of hiPSC cardiac spheroids. Nano Lett..

[117] Fleischer S, Shevach M, Feiner R, Dvir T (2014). Coiled fiber scaffolds embedded with gold nanoparticles improve the performance of engineered cardiac tissues. Nanoscale.

[118] Shevach M, Maoz BM, Feiner R, Shapira A, Dvir T (2013). Nanoengineering gold particle composite fibers for cardiac tissue engineering. J. Mater. Chem. B.

[119] You JO, Rafat M, Ye GJ, Auguste DT (2011). Nanoengineering the heart: conductive scaffolds enhance connexin 43 expression. Nano Lett..

[120] Navaei A (2016). Gold nanorod-incorporated gelatin-based conductive hydrogels for engineering cardiac tissue constructs. Acta Biomater..

[121] Martins AM (2014). Electrically conductive chitosan/carbon scaffolds for cardiac tissue engineering. Biomacromolecules.

[122] Shin SR (2013). Carbon-nanotube-embedded hydrogel sheets for engineering cardiac constructs and bioactuators. ACS Nano.

[123] Stout DA, Raimondo E, Marostica G, Webster TJ (2014). Growth characteristics of different heart cells on novel nanopatch substrate during electrical stimulation. Biomed. Mater. Eng..

