# Erratum: Restoring heart function and electrical integrity: Closing the circuit

**DOI:** 10.1038/s41536-017-0020-5

**Published:** 2017-09-06

**Authors:** Luís Miguel Monteiro, Francisco Vasques-Nóvoa, Lino Ferreira, Perpétua Pinto-do-Ó, Diana Santos Nascimento

**Affiliations:** 1 i3S – Instituto de Investigação e Inovação em Saúde, Universidade do Porto, Porto, Portugal; 2 INEB – Instituto de Engenharia Biomédica, Universidade do Porto, Porto, Portugal; 3 CNC – Center for Neuroscience and Cell Biology, Universidade de Coimbra, Coimbra, Portugal; 4 Departamento de Fisiologia e Cirurgia Cardiotorácica, Faculdade de Medicina da Universidade do Porto, Porto, Portugal; 5 ICBAS – Instituto de Ciências Biomédicas Abel Salazar, Universidade do Porto, Porto, Portugal

**Erratum to:** *npj Regen Med* (2017); doi:10.1038/s41536-017-0015-2; Published 07 April 2017

This Review Article contains typographical errors in the ‘Novel strategies to restore myocardial electrical conduction’ section under subheading ‘Gene therapies to treat arrhythmias’.

“Ex vivo optical mapping performed on whole rat hearts (both healthy or with MI) showed that patches reduced cardiac conduction velocities when applied on the epicardium. Thus, although patches exerted a negative effect on cardiac electrical integrity, they were successful in altering the electrophysiological properties of the tissue.”

Should read:

“Ex vivo optical mapping performed on whole rat hearts showed that patches reduced cardiac conduction velocities when applied on the epicardium of healthy hearts but increased in a heart with MI. Thus, patches were successful in altering the electrophysiological properties of the tissue.”

This Review Article also contains errors in Table 1. The correct Table 1 appears below as Table 1.

**Table 1:** Experimental results on the application of conductive materials in cardiac tissue engineering

Material	Refs.	Scaffold	Elastic modulus (kPa)/ Conductivity (S/m)	Cell source	Main results
Gold	[100]	AuNW-incorporated alginate scaffolds	~3.5/n.a.	Neonatal CM	Thick and aligned cell constructs; ↑ α-SA and Cx43; synchronous contractions
	[117]	AuNP-deposited PCL fibers	~60 × 10^3^/n.a.	Neonatal CM	Elongated CM, aligned and striated cell constructs; ↑ contraction rate and force
	[103]	AuNP-incorporated biodegradable PU scaffolds	~200–240/n.a.	H9C2	Improved cell spreading and alignment; ↑ Nkx2.5, ANF, NPPB expression
	[118]	AuNP-deposited PCL/gelatin scaffolds	n.a./n.a.	Neonatal CM	Elongated CM, visible striation and ↑ aspect ratio; ↑ contraction amplitudes and rates
	[101]	AuNP-deposited decellularized pig omental matrices	~12.5 × 10^3^/n.a.	Neonatal CM	Elongated CM, aligned and striated cell constructs; Cx43 between adjacent CM; ↑ contraction amplitude, calcium transient propagation velocity; ↓ excitation threshold
	[119]	AuNP-deposited thiol-HEMA/HEMA scaffolds	~600–1600/~11–15	Neonatal CM	CM presented as clusters or single cells; 2-fold ↑ Cx43 protein levels
	[120]	AuNW-incorporated GelMA hydrogels	~1.3/n.a.	Neonatal CM	↑ cell retention and viability; highly organized sarcomeric structures; ↑ beating frequency; more synchronous, stable, and robust beating behavior; synchronized calcium transients; ↓ excitation threshold
Carbon	[106]	MWCNT-embedded PG nanofibers	~373.5/n.a.	Neonatal CM	↑ CM alignment, metabolic activity and viability; ↑ Cx43 staining
	[104, 105]	MWCNT solution coating a glass substrate	n.a./n.a.	Neonatal CM	↑ metabolic activity; more negative membrane resting potential; ↑ αMHC, SERCA2a, Cx43; ↓ ANF
	[121]	Chitosan:CNTs composite scaffolds	~28.1/~0.25 (hydrated)	Neonatal CM	↑ CM alignment and metabolic activity; ↑ TnI, SERCA2a, GATA4, αMHC, Cx43, βMHC and ANF expression
	[107]	SWCNT-incorporated gelatin-chitosan hydrogels	~19.3 (175 p.p.m.)/n.a.	Neonatal CM	Concentration-dependent cytotoxicity; more developed sarcomeres; ↑ α-SA; intercellular Cx43 staining; ↑ beating rates and conduction velocity; ↓ AP duration
	[122]	CNT-embedded GelMA hydrogels	~20–54/n.a.	Neonatal CM	Aligned, interconnected CM; developed sarcomeres; attenuation of heptanol-induced intercellular coupling inhibition
	[123]	PLGA:CNFs composite substrates	n.a./~5 × 10^-4^–7 × 10^−3^	Human CM, rat EC, NIH/3T3	↑ CM density; ↓ ECs and fibroblast growth
	[109]	SWCNT/collagen solution coating a glass substrate	n.a./~1.90 × 10^-8^–1.77 × 10^3^	Neonatal CM	Marked striation and organized sarcomeres; functional beating syncytium; ↑TnI, Cx43, N-cadherin, plakophilin2 and plakoglobin expression; well-developed intercalated disc junctions; ↑ β1-integrin, FAK, p-ERK, MEF-2c and GATA4
	[108]	SWCNT-incorporated gelatin hydrogels	n.a. (shear modulus:~20–400 Pa)/~5 × 10^−5^	Neonatal CM	In vitro: aligned cell constructs; organized sarcomeres; ↑ α-SA and Cx43 levels; spontaneous electrical activity; In vivo (MI rats): ↑ Cx43, Na_V_1.5 and N-cadherin protein levels; unclear scaffold/scar boundary; presence of smooth-muscle cells and CD68^+^ macrophages; ↑ ejection fraction and fractional shortening. Electrical coupling was assessed by evaluating Cx43
Conductive polymers	[111]	PCL/PU blend scaffolds containing aniline pentamers	~1.3 × 10^3^/~10^-4^–10^4^	Neonatal CM	↑ TnT, Cx43, actinin-α-4
	[110]	Nanofibrous 2D meshes of HCl-doped PANI/PLGA blend	~91.7 × 10^3^/~0.31	Neonatal CM	Isolated cell clusters; spontaneous beating activity; ↑ TnI, Cx43 expression; intercellular Cx43 localization
	[112]	PPy/PCL/gelatin blend nanofibers on glass substrate	~16.8 × 10^3^/~1.3 × 10^−3^	Rabbit CM	↑ α-SA, TnT and Cx43; increasing PPy proportion disrupted mechanical properties and slowed CM growth
	[114]	Injectable PPy-grafted chitosan hydrogel	~2/~0.02	Neonatal CM	In vitro: ↑ Ca^2+^ transients velocity;In vivo (MI rats): QRS interval duration similar to healthy; ↑ transverse and border zone/scar region conduction velocities; ↑ ejection fraction, dP/dt max and min, preload recruitable stroke work; Note: although ex vivo optical mapping was performed to assess conduction velocities at the injection site, since the hydrogel was injected without cells, electrical coupling between implanted cells and the native myocardium was not evaluated
	[113]	Films of interpenetrating PPy and PCL networks	~9.3 × 10^5^/~0.10	HL-1	↑ proportion of cells with peripheral Cx43 expression;↑ Ca^2+^ transients velocity and spontaneous electrical activity frequency
	[115]	Films of chitosan and PANI	~6.7 × 10^3^/~16	n.a.	Ex vivo: rat cardiac slices-↓ transverse and longitudinal conduction velocities; Whole rat hearts (optical mapping) - ↓ conduction velocities on healthy hearts and ↑ on MI hearts.In vivo (healthy rats): unaffected ejection fraction, fractional shortening and no aggravated arrhythmia inducibility
Silicon	[116]	SiNW-incorporated cardiac cell spheroids	n.a./150–500	Neonatal CMs or hiPSC-derived CMs	Improved intercellular coupling (e.g., ↑ Cx43 and N-cadherin); improved contractile machinery development; ↑ β-MHC/α-MHC ratio; ↓ spontaneous beating frequency

*n.a*. non-available, *αMHC* alpha myosin heavy chain, *α-SA* alpha-sarcomericactinin, *βMHC* beta myosin heavy chain. *ANF* atrial natriuretic factor, *AuNP* gold nanoparticle, *AuNW* gold nanowire, *CM* cardiomyocyte, *CNT* carbon nanotube, *CNF* carbon nanofibers, *Cx43* connexin-43, *EC* endothelial cell, *ERK* extracellular-signal-regulated kinase, *ESC* embryonic stem cell, *FAK* focal adhesion kinase, *GelMA* gelatin methacrylate, *HEMA* hydroxyethylmethacrylate, *hiPSCs* human-induced pluripotent stem cells, *MEF-2c* myocyte-specific enhancer factor 2C, *MHC* myosin heavy chain, *MI* myocardial infarction, *MWCNT* multi-walled carbon nanotube, *NPPB* natriuretic peptide precursor B, *PANI* polyaniline, *PCL* polycaprolactone, *PECAM1* platelet endothelial cell adhesion molecule 1, *PLGA* polylactic-co-glycolic acid, *PPy* polypyrrole, *PU* polyurethane, *SERCA2a* sarcoplasmic reticulum Ca^2+^ ATPase 2a, *SiNW* silicon nanowire, *SWCNT* single-walled carbon nanotube, *TnI* troponin I, *TnT* troponin

